# Socioeconomic inequalities in psychosocial well-being among adolescents under the COVID-19 pandemic: a cross-regional comparative analysis in Hong Kong, mainland China, and the Netherlands

**DOI:** 10.1007/s00127-024-02649-7

**Published:** 2024-04-04

**Authors:** Gary Ka-Ki Chung, Xiaoting Liu, Karlijn Massar, Karen Schelleman-Offermans, Hans Bosma, Yat-Hang Chan, Siu-Ming Chan, Ji-Kang Chen, Hung Wong, Roger Yat-Nork Chung

**Affiliations:** 1 CUHK Institute of Health Equity, The Chinese University of Hong Kong, Hong Kong, China; 2 JC School of Public Health and Primary Care, Faculty of Medicine, The Chinese University of Hong Kong, Hong Kong, China; 3 School of Public Affairs, Zhejiang University, Hangzhou, China; 4 Department of Work and Social Psychology, Faculty of Psychology and Neuroscience, Maastricht University, P.O. Box 616, Maastricht, 6200 MD The Netherlands; 5 Department of Social Medicine, Faculty of Health, Medicine and Life Sciences, Maastricht University, P.O. Box 616, Maastricht, 6200 MD The Netherlands; 6 CityU Department of Social and Behavioural Sciences, The City University of Hong Kong, Hong Kong, China; 7 Department of Social Work, The Chinese University of Hong Kong, Hong Kong, China; 8 CUHK Centre for Bioethics, The Chinese University of Hong Kong, Hong Kong, China

**Keywords:** Adolescents, COVID-19, Socioeconomic inequality, Psychosocial well-being, Cross-regional, Mediator

## Abstract

**Background:**

Despite evidence on socioeconomic inequalities in psychosocial well-being of adolescents under the COVID-19 pandemic, the explanatory factors and their potential variations across contexts remained understudied. Hence, this cross-regional study compared the extent of inequalities and the mediating pathways across Hong Kong, Mainland China, and the Netherlands.

**Methods:**

Between July 2021 and January 2022, 25 secondary schools from diverse socioeconomic background were purposively sampled from Hong Kong, Zhejiang (Mainland China), and Limburg (the Netherlands). 3595 junior students completed an online survey during class about their socioeconomic position, psychosocial factors, and well-being. Socioeconomic inequalities were assessed by multiple linear regressions using the Slope Index of Inequality (SII), whereas the mediating pathways through learning difficulty, overall worry about COVID-19, impact on family’ financial status, resilience, trust in government regarding pandemic management, and adaptation to social distancing were examined by mediation analyses moderated by regions.

**Results:**

The adverse psychosocial impact of COVID-19 was stronger in the Netherlands and Hong Kong compared with Mainland China. The greatest extent of socioeconomic inequalities in the change in psychosocial well-being was observed among students in the Netherlands (SII = 0.59 [95% CI = 0.38–0.80]), followed by Hong Kong (SII = 0.37 [0.21–0.52]) and Mainland China (SII = 0.12 [0.00–0.23]). Learning difficulty and resilience were the major mediators in Mainland China and Hong Kong, but to a lesser extent in the Netherlands.

**Conclusion:**

Socioeconomic inequalities in psychosocial well-being were evident among adolescents under the pandemic, with learning difficulty and resilience of students as the key mediators. Differences in the social contexts should be considered to better understand the variations in inequalities and mediating pathways across regions.

## Introduction

Adolescents have been facing significant challenges due to the COVID-19 pandemic during their critical developmental period. Although adolescents are not particularly vulnerable to the SARS-CoV-2 virus itself, an extensive body of research revealed deterioration in their mental health and psychosocial well-being under partial or full lockdowns and other stringent containment measures [1]. Notably, such psychosocial impact affects disadvantaged adolescents more severely in general due to a lack of resources and opportunities to cope with pandemic-related stressors [2–5]. Previous studies showed that greater learning difficulties under school closure, worries about infection and the financial status of their family, more disrupted social interactions that hinder the establishment of peer and intimate relationships during adolescence, and worse attitudes towards government and its pandemic responses, were plausible reasons for the disproportionate psychosocial impact on adolescents across the socioeconomic ladder [6–11]. On the other hand, resilience (i.e., the ability to bounce back from adversity) acted as a protective psychosocial factor to buffer against these stressors [12, 13].

Despite the solid evidence on the existence of socioeconomic inequalities in psychosocial well-being among adolescents, such disproportionate psychosocial impact may vary across world regions. As highlighted by Barn et al. [14], research on mental health inequalities on children or adolescents under COVID-19 is highly sensitive to social context, in which their experience under the pandemic is not only linked to their socioeconomic position but also the varying public reception and policy responses to COVID-19 across regions. While relevant studies on adolescents are scarce, the current international literature on adults may provide some clues on the important contextual factors that shape the extent of inequalities in psychosocial well-being. For example, Maffly-Kipp et al. [15] reported a greater extent of mental health inequalities in regions with more severe COVID-19 outbreaks. Lee et al. [16] also concluded a less severe psychosocial impact of COVID-19 in regions where swift government responses with stringent containment measures were in place, which could potentially protect the disadvantaged who were at greater mental health risk. Moreover, the welfare system and provision of social security benefits may affect population mental health and its associated inequalities under COVID-19 [17], although inequalities may not necessarily be smaller in regions with more comprehensive welfare regimes [18, 19]. To further explore the generalizability of these potential contextual effects, cross-regional studies on adolescents are warranted to compare the psychosocial impact of COVID-19 and the extent of socioeconomic inequalities in psychosocial well-being across world regions with diverse social contexts. By examining these regional differences, researchers and policymakers will be able to gain a deeper understanding of how specific pandemic responses and underlying societal features contribute to variations in psychosocial outcomes, which will in turn facilitate a more comprehensive interpretation of the psychosocial impact of the pandemic and inform potential policies and intervention approaches to better support adolescents in need.

In the present study, we included Hong Kong, Mainland China, and the Netherlands for comparison with reference to the above-mentioned contextual factors identified in the general adult population. First, the severity of outbreak varied as the COVID-19 incidence and mortality rates were low in both Hong Kong and Mainland China during the study period but apparently more severe in the Netherlands. Second, the stringency of containment measures was the greatest in Mainland China, followed by Hong Kong and the lowest in the Netherlands, which did not match with the corresponding severity of outbreak. Furthermore, the Netherlands has a more advanced welfare system than Hong Kong and Mainland China, which may have implications on the resultant inequalities under the pandemic. Therefore, the present study aimed to (i) assess the extent of socioeconomic inequalities in psychosocial factors and outcomes among secondary school students under the pandemic, (ii) delineate the potential mediating roles of psychosocial factors in any observed socioeconomic inequalities in psychosocial outcomes, and (iii) explore how the above inter-relationships might vary across regions with different social contexts.

## Methods

### Study population

Data were collected from a purposive sample of secondary schools separately in Hong Kong (a highly developed special administrative region of China), Zhejiang (a relatively developed eastern coastal province in Mainland China), and Limburg (the most southern province with considerable socioeconomic differences and worse population health in the Netherlands). A maximum variation sampling approach was adopted to ensure a diverse spectrum of socioeconomic background of the participating schools, which was selected based on an index of economic, social, and cultural status defined by the Programme for International Student Assessment, school type (i.e., public or private), or location (i.e., urban or rural) depending on the socioeconomic stratification system of each region. Junior secondary school students in selected grades and classes, who agreed to participate and had consent from their parents or guardians, were invited to complete an online survey during class. In total, 3,595 students were recruited from 25 secondary schools across the three regions between July 2021 and January 2022, with 1,095 from twelve schools in Hong Kong, 2,014 from nine schools in Zhejiang, and 486 from four schools in Limburg. The corresponding response rates were 85.5%, 92.0%, and 95.1%, respectively.

### Measurements

Information on students’ socioeconomic position, psychosocial outcomes, a range of psychosocial factors, and other socio-demographic factors was collected via the online survey. The questionnaire with the same set of questions was translated into traditional Chinese, simplified Chinese, and Dutch for survey administration in Hong Kong, Zhejiang, and Limburg, respectively, by native bilingual researchers. Detailed measurements of variables of interest are listed below.

#### Socioeconomic position

The socioeconomic position of students was assessed based on the social ladder measure of the MacArthur Scale of Subjective Social Status – Youth Version [20]. Students were asked to mark the rung that best represented where their family would be on a socioeconomic ladder ranging from 1 (i.e., the worst off) to 10 (i.e., the best off). We adopted the MacArthur Scale of Subjective Social Status – Youth Version because a previous systematic review showed that it is most strongly associated with health outcomes related to psychological processes [21], and showed its superior role over objective socioeconomic measures in predicting health outcomes such as self-rated health, depression, and well-being among adolescents [21].

#### Psychosocial outcomes

To assess the change in psychosocial well-being before and during the pandemic, respondents were asked how much more/less (i) relaxed, (ii) confident about future, (iii) cheerful, (iv) anxious/stressed, and (v) hopeless they felt as compared with the time before COVID-19, with five ordinal options recoded from − 2 (i.e., much less) to 2 (i.e., much more) with a neutral point at 0 indicating no apparent change. The last two items were reversely coded for analysis to consistently show the results in one direction, where a positive mean score represents better psychosocial well-being as compared with that before the pandemic. The five selected items were adopted and modified from the COVID-19 Adolescent Symptom & Psychological Experience Questionnaire [22], with an internal reliability of 0.782 (0.769 in Hong Kong; 0.791 in Zhejiang; 0.690 in Limburg). Also, the mental health status of students during the pandemic was measured by the revised Mental Health Inventory-5 [23] with a total score ranging from 0 to 15, where a higher score represents a better mental health status under COVID-19. The internal reliability of the revised Mental Health Inventory-5 was acceptable at 0.653 (0.619 in Hong Kong; 0.583 in Zhejiang; 0.776 in Limburg). Moreover, loneliness was measured using the 3-item Loneliness Scale [24] on (i) feeling that you lack companionship, (ii) feeling left out, and (iii) feeling isolated from others, each with three ordinal options (i.e., 1 = hardly ever; 2 = some of the time; 3 = often). A higher score represents a greater level of loneliness. The internal reliability of the Loneliness Scale was satisfactory at 0.870 (0.897 in Hong Kong; 0.859 in Zhejiang; 0.783 in Limburg).

#### Psychosocial factors

In addition to the psychosocial outcomes, six psychosocial factors, deemed as the social conditions or social determinants of psychosocial well-being that are particularly affected by the COVID-19 pandemic, were also adopted including (i) resilience, (ii) learning difficulty, (iii) overall worry about COVID-19, (iv) impact on family’s financial status, (v) trust in government regarding pandemic management, and (vi) adaptation to social distancing. First, resilience was measured using the 6-item Brief Resilience Scale, which assesses specifically the ability to bounce back or recover from adversities and to cope with health-related stressors [25]. Responses were rated on a 5-point Likert scale from ‘strongly disagree’ to ‘strongly agree’, with a mean score ranging from 1 to 5; a higher score represents a greater level of resilience. The internal reliability of the Brief Resilience Scale was acceptable at 0.704 (0.781 in Hong Kong; 0.677 in Zhejiang; 0.501 in Limburg). Second, as for learning difficulty, students were asked to what extent they experienced the following problems including (i) access to a digital device when needed, (ii) internet access, (iii) finding a quiet place to study, (iv) understanding school assignments, and (v) finding someone who could help with schoolwork, each with four ordinal options (i.e., 1 = never; 2 = sometimes; 3 = often; 4 = always), with a mean score ranging from 1 to 4; a higher score represents a greater level of learning difficulty. These items were selected with reference to the PISA Global Crises Questionnaire Module, a tool with an internal reliability of 0.769 (0.767 in Hong Kong; 0.783 in Zhejiang; 0.660 in Limburg) to capture learning experiences during COVID-19 [26]. Third, regarding overall worry about COVID-19, students were asked how worried they were about the local COVID-19 situation with five ordinal options (i.e., 1 = not at all; 2 = slightly; 3 = moderately; 4 = very; 5 = extremely). Fourth, as for the impact on family’s financial status, students were asked to what extent the changes related to the COVID-19 outbreak had created financial problems for their family with five ordinal options (i.e., 1 = not at all; 2 = slightly; 3 = moderately; 4 = very; 5 = extremely). Fifth, regarding trust in government regarding pandemic management [27], students were asked “how much do you trust the government to take care of its citizens during the COVID-19 pandemic?” with five ordinal options, recoded from − 2 (i.e., distrust completely) to 2 (i.e., trust completely) with a neutral point at 0 indicating “neither trust nor distrust.” Last, as for the adaptation to social distancing, students were asked whether they complied well to the social distancing measures implemented by the local government with five ordinal options, recoded from − 2 (i.e., strongly disagree) to 2 (i.e., strongly agree) with a neutral point at 0.

#### Contextual information across regions

Background information in Hong Kong, Zhejiang, and Limburg including GDP per capita [28–30], inequality-adjusted Human Development Index [31], Gini coefficient [32, 33], population density [34–36], COVID-19 severity [37–39] were collected from the World Bank, Human Development Reports from the United Nations Development Programme, and local government statistics in 2021 or the latest available year. The stringency of their containment measures across the three regions was obtained from the Oxford COVID-19 Government Response Tracker [40]. As the containment measures may vary over time, a weighted average of the stringency index over the data collection period in each region was used.

### Statistical analysis

Descriptive statistics of the basic characteristics of students in terms of age group, gender, ethnicity, household size, and socioeconomic position were derived using count data with percentages, whereas that of the psychosocial outcomes and factors were reported based on their mean scores with standard deviations (SD). Chi-squared tests and analysis of variance (ANOVA) tests were used to examine the difference in characteristics and mean scores across the three regions. The Slope Index of Inequality (SII) [41] was employed to assess the extent of socioeconomic inequalities in psychosocial outcomes and factors in absolute terms. To measure the SII, a fractional rank score, scaled from 0 (i.e., lowest along the socioeconomic ladder) to 1 (i.e., highest along the socioeconomic ladder), was first calculated based on the distribution of socioeconomic position of students in each region. The resultant fractional rank score was then adopted as the independent variable in separate multivariable linear regression models to estimate the SII for each psychosocial outcome and factor, with adjustments for age, gender, ethnicity, and household size. Regarding the interpretation, SII above 0 represents inequality with a higher mean score of the psychosocial outcomes or factors, or a greater increase/smaller decrease in pandemic-related changes in psychosocial well-being, in the most advantaged as compared with the least advantaged along the socioeconomic ladder. Likelihood ratio test between nested models with and without an interaction term of the fractional rank score of socioeconomic position by region was employed to examine whether the extent of socioeconomic inequalities in psychosocial outcomes and factors differed across regions. Moreover, to assess the potential mediating roles of the psychosocial factors in the associations of socioeconomic position with the three psychosocial outcomes, moderated mediation analyses were performed to delineate the direct effect and indirect effects via the psychosocial factors based on seemingly unrelated regression models. It was assumed that region would moderate both the paths between socioeconomic position and psychosocial factors and the paths between the psychosocial factors and each outcome. To explore the sensitivity of mediation effects to unmeasured confounding (i.e., the sequential ignorability assumption), sensitivity analyses on the major indirect pathways by each region were conducted based on the sensitivity parameter, defined as the correlation between the residuals from the mediator and outcome regression models, at which the average causal mediation effect equal zero. Multicollinearity among psychosocial outcomes and factors was also assessed based on variance inflation factor at the threshold of 5. Listwise deletion was adopted to handle missing data due to incomplete responses. In total, there were 1020, 2005, and 457 complete respondents in Hong Kong, Zhejiang, and Limburg, respectively. All data analyses were conducted using Stata version 14 and R version 4.3.1, where all statistical tests were two-tailed with a significance level of *p* < 0.05, except that a significance level of *p* < 0.1 was used for interaction tests due to lower statistical power in regressions with interaction terms [42].

## Results

### Descriptive statistics of respondents across regions

The general socio-demographic background as well as the severity of COVID-19 and the stringency of related containment measures in Hong Kong, Zhejiang, and Limburg are depicted in Table 1. Generally speaking, the GDP per capita and inequality-adjusted Human Development Index were higher in Hong Kong and Limburg than in Zhejiang, while Hong Kong had the greatest level of income inequality and population density of all the study regions. Over the study period, the daily numbers of COVID-19 infections and deaths were the highest in Limburg, far exceeding the corresponding figures in Hong Kong and Zhejiang. Regarding the COVID-19 containment measures, the greatest stringency was found in Zhejiang, followed by Hong Kong and the lowest in Limburg.

**Table 1:** Summary of background information, COVID-19 situation, and containment measures across regions

	Hong Kong	Zhejiang, Mainland China	Limburg, the Netherlands
*General background (by the end of 2021 or latest available)*			
GDP per capita (in USD)	49,629 ^a^	17,520 ^b^	52,012 ^c^
Inequality-adjusted Human Development Index	0.824	0.651 ^d^	0.878 ^d^
Gini coefficient	0.539 (in 2016)	0.382 (in 2019) ^d^	0.292 (in 2019) ^d^
Population density (person per km^2^)	6800	620	520
*Data collection period*	20 Sept 2021–26 Oct 2021	16 Jan 2022–22 Jan 2022	1 Jul 2021–22 Oct 2021
*COVID-19 situation during data collection period*			
Average daily number of cases per million	0.62	0.06	17,464
Average daily number of deaths per million	0	0	0.48
*Containment measures during the period of data collection*			
Average stringency Index	59.26	79.17 ^﻿d^	41.08﻿ ﻿^d^

^a^ HK$387,110 at the average linked exchange rate of 7.800 to USD$1 in 2021

^b^ RMB$ 113,032 at the average exchange rate of 6.452 to USD$1 in 2021

^c^ €43,992 at that average exchange rate of 0.846 to USD$1 in 2021

^d^ Figures at country level rather than region level

The basic characteristics of respondents in terms of age group, gender, ethnicity, household size, and socioeconomic position are presented in Table 2. Significant results for all chi-squared tests suggested that there were differences in sample characteristics across regions. Apart from demographic factors, the level of psychosocial outcomes and factors under COVID-19 are also illustrated in Table 3. Specifically, the overall change in psychosocial well-being due to COVID-19 tended to be positive in Zhejiang (mean = 0.42 ± 0.76) but almost neutral in Hong Kong (mean = 0.02 ± 0.72) and Limburg (mean = -0.02 ± 0.65). Also, mental health status during COVID-19 was the best among students in Zhejiang (mean = 11.37 ± 2.44), followed by Limburg (mean = 10.85 ± 2.57), while it was the worst in Hong Kong (mean = 9.55 ± 2.47). In addition, students in Zhejiang had lower level of loneliness (mean = 3.69 ± 1.27), as compared with students in Hong Kong (mean = 4.50 ± 1.88) and Limburg (mean = 4.65 ± 1.71). Regarding the psychosocial factors, students in Zhejiang performed better in general with greater resilience, lower learning difficulty, greater trust in government regarding pandemic management, and better adaptation to social distancing. On the contrary, students in Hong Kong tended to have greater overall worry about COVID-19, higher financial impact on their family, and lower trust in government response to COVID-19, whereas students in Limburg tended to have lower level of resilience and greater learning difficulty during school closure, despite a lower financial impact on their family. Significant results for all ANOVA tests suggested that there were differences in levels of psychosocial outcomes and factors under COVID-19 across regions.

**Table 2:** Basic characteristics of sampled students across regions

	Hong Kong (*N* = 1095)	Zhejiang, Mainland China (*N* = 2014)	Limburg, the Netherlands (*N* = 486)	
	N (%)	N (%)	N (%)	*p*_chi−squared_
*Age* ^a^				< 0.001
13 or below	0 (0.0)	992 (49.3)	219 (45.1)	
14	903 (82.5)	694 (34.5)	195 (40.1)	
15	154 (14.1)	290 (14.4)	57 (11.7)	
16	38 (3.5)	32 (1.6)	13 (2.7)	
17 or above	0 (0.0)	6 (0.3)	2 (0.4)	
*Gender* ^b^				< 0.001
Female	514 (47.0)	1027 (51.2)	217 (44.7)	
Male	580 (53.0)	978 (48.8)	257 (52.9)	
Others	0 (0.0)	0 (0.0)	12 (2.5)	
*Ethnicity* ^c^				< 0.001
Ethnic Chinese	1054 (96.4)	1967 (97.7)	8 (1.6)	
Other Asians	15 (1.4)	2 (0.1)	1 (0.2)	
Black / African / Caribbean	2 (0.2)	0 (0.0)	4 (0.8)	
White	5 (0.5)	12 (0.6)	455 (93.6)	
Mixed / multiple ethnic groups	13 (1.2)	5 (0.2)	6 (1.2)	
Others	4 (0.4)	28 (1.4)	12 (2.5)	
*Household size* ^d^				< 0.001
1	15 (1.5)	19 (0.9)	1 (0.2)	
2	61 (5.9)	142 (7.1)	15 (3.3)	
3	228 (22.2)	644 (32.0)	73 (15.9)	
4	398 (38.8)	654 (32.5)	214 (46.7)	
5	217 (21.1)	275 (13.7)	115 (25.1)	
6 or above	108 (10.5)	280 (13.9)	40 (8.7)	
*Socioeconomic position* ^e^				< 0.001
1	22 (2.1)	30 (1.5)	3 (0.6)	
2	16 (1.6)	49 (2.4)	0 (0.0)	
3	75 (7.3)	102 (5.1)	0 (0.0)	
4	138 (13.4)	196 (9.8)	8 (1.7)	
5	327 (31.7)	588 (29.3)	20 (4.3)	
6	213 (20.7)	403 (20.1)	66 (14.2)	
7	134 (13.0)	329 (16.4)	122 (26.2)	
8	69 (6.7)	191 (9.5)	140 (30.0)	
9	17 (1.7)	46 (2.3)	62 (13.3)	
10	19 (1.8)	71 (3.5)	45 (9.7)	

Missing values in Hong Kong: ^a^ 0; ^b^ 1; ^c^ 2; ^d^ 68; ^e^ 65

Missing values in Zhejiang: ^a^ 0; ^b^ 9; ^c^ 0; ^d^ 0; ^e^ 9

Missing values in Limburg: ^a^ 0; ^b^ 0; ^c^ 0; ^d^ 28; ^e^ 20

**Table 3:** Levels of psychosocial outcomes and their related factors under COVID-19 across regions

	Hong Kong	Zhejiang, Mainland China	Limburg, the Netherlands	
	Mean ± SD	Mean ± SD	Mean ± SD	*p*_ANOVA_
*Psychosocial outcomes*				
Change in psychosocial well-being due to COVID-19 ^a^	0.02 ± 0.72	0.42 ± 0.76	-0.02 ± 0.65	< 0.001
Mental health status during COVID-19 ^b^	9.55 ± 2.47	11.37 ± 2.44	10.85 ± 2.57	< 0.001
Loneliness during COVID-19 ^c^	4.50 ± 1.88	3.69 ± 1.27	4.65 ± 1.71	< 0.001
*Psychosocial factors*				
Resilience ^*d*^	3.15 ± 0.69	3.44 ± 0.67	2.99 ± 0.63	< 0.001
Learning difficulty ^e^	1.66 ± 0.57	1.40 ± 0.48	1.80 ± 0.48	< 0.001
Overall worry about COVID-19 ^f^	2.42 ± 1.02	2.08 ± 0.98	2.03 ± 1.03	< 0.001
Impact on family’s financial status ^g^	2.26 ± 0.99	2.01 ± 0.90	1.27 ± 0.63	< 0.001
Trust in government regarding pandemic management ^h^	-0.33 ± 0.99	1.23 ± 1.13	0.20 ± 1.19	< 0.001
Adaptation to social distancing ^i^	0.50 ± 0.97	1.12 ± 0.96	0.46 ± 1.25	< 0.001

Missing values in Hong Kong: ^a^ 17; ^b^ 11; ^c^ 37; ^d^ 47; ^e^ 23; ^f^ 64; ^g^ 75; ^h^ 52; ^i^ 50

Missing values in Zhejiang: ^a^ 9; ^b^ 9; ^c^ 9; ^d^ 9; ^e^ 9; ^f^ 9; ^g^ 9; ^h^ 9; ^i^ 9

Missing values in Limburg: ^a^ 1; ^b^ 0; ^c^ 7; ^d^ 10; ^e^ 4; ^f^ 20; ^g^ 29; ^h^ 17; ^i^ 17

### Extent of socioeconomic inequalities in psychosocial outcomes and factors across regions

As shown in Table 4, students of lower socioeconomic position fared worse in almost all psychosocial outcomes and factors, with substantial heterogeneity in the extent of inequalities across the three regions. In terms of the change in psychosocial well-being, the extent of inequality was the lowest among students in Zhejiang (SII = 0.12; 95% CI = 0.00–0.23; *p* = 0.054), but higher among students in Hong Kong (SII = 0.37; 95% CI = 0.21–0.52; *p* < 0.001) and in Limburg (SII = 0.59; 95% CI = 0.38–0.80; *p* < 0.001), with a significant interaction effect (*p* = 0.003). Similar pattern was observed for mental health status and loneliness during COVID-19, where the extent of inequalities was the lowest in Zhejiang and the greatest in Limburg (*p* = 0.001 and 0.040, respectively, for interaction effects). As for psychosocial factors, significant socioeconomic inequalities in learning difficulty, impact on family’s financial status, and resilience were consistently observed across regions (all *p* < 0.001, except for resilience in Limburg with *p* = 0.024). Specifically in Zhejiang, significant socioeconomic inequalities were observed for all the factors (*p* < 0.001), where the extent of inequalities in overall worry about COVID-19, financial impact on their family, and adaptation to social distancing was the greatest across the three regions. In addition, significant socioeconomic inequality in trust in government regarding pandemic management was also observed in Hong Kong (*p* = 0.014), with lower trust in government observed in adolescents with a low SEP compared with a high SEP. Significant interaction effects between fractional rank of socioeconomic position and region were observed for overall worry about COVID-19 (*p* = 0.007), adaptation to social distancing (*p* < 0.001), and marginally for financial impact on their family (*p* = 0.078) and resilience (*p* = 0.076).

**Table 4:** Extent of absolute socioeconomic inequalities in psychosocial outcomes and their related factors under COVID-19 across regions

	Hong Kong		Zhejiang, Mainland China		Limburg, the Netherlands		
	SII [95% CI] ^a^	*p*-value	SII [95% CI] ^a^	*p*-value	SII [95% CI] ^a^	*p*-value	*p*_interaction_^b^
*Psychosocial outcomes*							
Change in psychosocial well-being due to COVID-19	0.37 [0.21, 0.52]	< 0.001	0.12 [0.00, 0.23]	0.054	0.59 [0.38, 0.80]	< 0.001	0.003
Mental health status during COVID-19	1.60 [1.07, 2.13]	< 0.001	1.13 [0.75, 1.50]	< 0.001	2.69 [1.89, 3.49]	< 0.001	0.001
Loneliness during COVID-19	-0.87 [-1.27, -0.46]	< 0.001	-0.57 [-0.76, -0.37]	< 0.001	-1.25 [-1.81, -0.68]	< 0.001	0.040
*Psychosocial factors*							
Learning difficulty	-0.28 [-0.40, -0.15]	< 0.001	-0.20 [-0.28, -0.13]	< 0.001	-0.33 [-0.48, -0.18]	< 0.001	0.331
Overall worry about COVID-19	-0.09 [-0.32, 0.13]	0.406	-0.52 [-0.67, -0.37]	< 0.001	-0.13 [-0.48, 0.21]	0.445	0.007
Impact on family’s financial status	-0.54 [-0.76, -0.33]	< 0.001	-0.77 [-0.91, -0.64]	< 0.001	-0.46 [-0.66, -0.27]	< 0.001	0.078
Resilience	0.51 [0.37, 0.66]	< 0.001	0.38 [0.28, 0.48]	< 0.001	0.24 [0.03, 0.44]	0.024	0.076
Trust in government regarding pandemic management	0.27 [0.06, 0.49]	0.014	0.42 [0.24, 0.59]	< 0.001	0.06 [-0.32, 0.45]	0.747	0.157
Adaptation to social distancing	0.11 [-0.10, 0.33]	0.292	0.48 [0.33, 0.62]	< 0.001	-0.26 [-0.67, 0.15]	0.222	< 0.001

^a^ Adjusted for age, gender, ethnicity, and household size in separate multivariable linear regression models against each psychosocial outcome or factor

^b^*p*-value of interaction term between fractional rank of socioeconomic position and region

SII: Slope index of inequality

**Table 5:** Moderated mediation analyses for the socioeconomic inequalities in psychosocial outcomes under COVID-19 across regions

			Hong Kong		Zhejiang, Mainland China		Limburg, the Netherlands	
			β [95% CI] ^a^	*p*-value	β [95% CI] ^a^	*p*-value	β [95% CI] ^a^	*p*-value
*Change in psychosocial well-being due to COVID-19*								
Direct effect			0.09 [-0.07, 0.24]	0.266	-0.07 [-0.18, 0.04]	0.227	0.48 [0.25, 0.71]	< 0.001
Total indirect effect			0.26 [0.19, 0.34]	< 0.001	0.19 [0.14, 0.24]	< 0.001	0.08 [-0.02, 0.17]	0.116
Specific indirect effects								
* Learning difficulty*			0.06 [0.03, 0.09]	< 0.001	0.03 [0.01, 0.05]	0.001	0.05 [0.00, 0.11]	0.052
* Overall worry about COVID-19*			0.01 [-0.01, 0.02]	0.391	0.01 [-0.01, 0.02]	0.391	0.01 [-0.02, 0.05]	0.344
* Impact on family’s financial status*			0.02 [-0.01, 0.04]	0.248	0.01 [-0.02, 0.04]	0.447	-0.06 [-0.12, 0.01]	0.074
* Resilience*			0.17 [0.11, 0.23]	< 0.001	0.10 [0.07, 0.14]	< 0.001	0.04 [-0.01, 0.09]	0.107
* Trust in government regarding pandemic management*			0.02 [0.00, 0.03]	0.123	0.01 [0.00, 0.02]	0.138	0.00 [-0.02, 0.02]	0.790
* Adaptation to social distancing*			0.00 [-0.01, 0.00]	0.582	0.03 [0.01, 0.05]	0.001	0.02 [-0.01, 0.04]	0.164
*Mental health status during COVID-19*								
Direct effect			0.56 [0.08, 1.04]	0.022	0.17 [-0.18, 0.51]	0.341	1.89 [1.17, 2.61]	< 0.001
Total indirect effect			1.03 [0.74, 1.32]	< 0.001	0.99 [0.80, 1.19]	< 0.001	0.89 [0.41, 1.37]	< 0.001
Specific indirect effects								
* Learning difficulty*			0.14 [0.05, 0.23]	0.002	0.14 [0.08, 0.21]	< 0.001	0.52 [0.22, 0.83]	0.001
* Overall worry about COVID-19*			0.02 [-0.03, 0.07]	0.390	0.10 [0.04, 0.16]	0.001	0.08 [-0.08, 0.25]	0.326
* Impact on family’s financial status*			0.09 [0.00, 0.17]	0.050	0.20 [0.11, 0.29]	< 0.001	0.00 [-0.16, 0.16]	0.988
* Resilience*			0.75 [0.51, 0.99]	< 0.001	0.42 [0.30, 0.55]	< 0.001	0.21 [-0.03, 0.45]	0.089
* Trust in government regarding pandemic management*			0.04 [-0.01, 0.09]	0.146	0.06 [0.02, 0.10]	0.007	0.01 [-0.05, 0.07]	0.790
* Adaptation to social distancing*			-0.01 [-0.03, 0.01]	0.519	0.07 [0.02, 0.13]	0.009	0.07 [-0.02, 0.15]	0.136
*Loneliness during COVID-19*								
Direct effect			-0.15 [-0.45, 0.16]	0.351	-0.22 [-0.44, 0.00]	0.053	-0.73 [-1.19, -0.26]	0.002
Total indirect effect			-0.73 [-0.93, -0.53]	< 0.001	-0.35 [-0.45, -0.24]	< 0.001	-0.51 [-0.79, -0.24]	< 0.001
Specific indirect effects								
* Learning difficulty*			-0.24 [-0.34, -0.13]	< 0.001	-0.16 [-0.22, -0.09]	< 0.001	-0.29 [-0.46, -0.11]	0.001
* Overall worry about COVID-19*			-0.01 [-0.02, 0.01]	0.453	-0.02 [-0.05, 0.01]	0.241	-0.04 [-0.13, 0.04]	0.331
* Impact on family’s financial status*			-0.05 [-0.10, 0.01]	0.076	-0.02 [-0.08, 0.03]	0.417	-0.03 [-0.13, 0.07]	0.543
* Resilience*			-0.42 [-0.56, -0.28]	< 0.001	-0.14 [-0.19, -0.09]	< 0.001	-0.10 [-0.21, 0.02]	0.100
* Trust in government regarding pandemic management*			-0.03 [-0.06, 0.01]	0.141	-0.02 [-0.04, 0.01]	0.184	-0.01 [-0.08, 0.06]	0.789
* Adaptation to social distancing*			0.01 [-0.02, 0.03]	0.452	0.01 [-0.02, 0.04]	0.587	-0.05 [-0.11, 0.01]	0.122

^a^ Moderated mediation models included the fractional rank of socioeconomic position, age, gender, ethnicity, household size, psychosocial factors, as well as region and the associated interaction terms between socioeconomic position and region and between psychosocial factors and region

## Moderated mediating roles in the socioeconomic inequalities in psychosocial outcomes across regions

As presented in Table 5, results of moderated mediation showed that the effect of socioeconomic position on psychosocial outcomes was mainly operated through the indirect pathways in Hong Kong and Zhejiang, while in Limburg the direct effect was generally stronger than the indirect effect. Taking the change in psychosocial well-being due to COVID-19 as an example, the direct effect of socioeconomic position was only significant in Limburg (β = 0.48; 95% CI = 0.25–0.71; *p* < 0.001) but not in Hong Kong (β = 0.09; 95% CI = -0.07–0.24; *p* = 0.266) and Zhejiang (β = 0.07; 95% CI = -0.18–0.04; *p* = 0.227), whereas the total indirect effect via psychosocial factors were significant in both Hong Kong (β = 0.26; 95% CI = 0.19–0.34; *p* < 0.001) and Zhejiang (β = 0.19; 95% CI = 0.14–0.24; *p* < 0.001) but not in Limburg (β = 0.08; 95% CI = -0.02–0.17; *p* = 0.116). Similar patterns applied to mental health status and loneliness during COVID-19, except that the direct effect on mental health was also significant in Hong Kong and that both the direct and indirect effects on mental health status and loneliness were all significant in Limburg. As for the specific indirect paths, learning difficulty and resilience were consistently the strongest mediators in Hong Kong, Zhejiang, and, to a lesser extent, Limburg. Sensitivity analyses on the two major indirect pathways via learning difficulty and resilience against each of the three psychosocial outcomes in each region showed that the sensitivity parameters at which the average causal mediation effect equal zero were above 0.25 in most cases and up to 0.35 for half of the cases, indicating the robustness of these major mediation effects to unmeasured confounding. Multicollinearity among psychosocial outcomes and factors was minimal as their variance inflation factors, ranging from 1.09 to 1.64, were all below the threshold of 5.

## Discussion

The present cross-regional comparative study showed that students in Mainland China generally fared better than those in Hong Kong and the Netherlands during the COVID-19 pandemic under study, with even an indication of a positive change in psychosocial well-being and better overall scores in most psychosocial outcomes and factors on average. Although the overall change in psychosocial well-being in Hong Kong and the Netherlands was almost neutral, this finding should be interpreted with the observed significant socioeconomic inequality being taken into account, which showed that psychosocial well-being among the socioeconomically disadvantaged students have generally worsened, while their advantaged counterparts were more likely to have experienced a positive change during the study period. Specifically, students in the Netherlands had the greatest extent of socioeconomic inequalities in psychosocial outcomes, whereas students in Mainland China had the lowest extent despite greater inequalities in psychosocial factors. After considering the mediating roles of psychosocial factors, the direct effects of socioeconomic position on psychosocial outcomes were substantially mitigated in Hong Kong and Mainland China but to a lesser extent in the Netherlands. This implied that the psychosocial factors included in the present study, in particular learning difficulty and resilience, were able to explain the majority of the associations between socioeconomic position and psychosocial outcomes only among students in Hong Kong and Mainland China.

The better overall performance among students in Mainland China could plausibly be attributed to its lowest local spread and severity of COVID-19 outbreaks across the three regions during the study period. Despite the high overall stringency of containment measures, schools in Zhejiang remained largely open for face-to-face learning except for the postponed semester commencement due to the first outbreak in early 2020 [43] and school closure for less than one month due to the second outbreak in late December 2021 [44]. Therefore, the duration of school closure due to regional lockdown in Zhejiang was much shorter than in Hong Kong and the Netherlands, where several waves of prolonged school closures in response to outbreaks had been imposed between 2020 and 2021 [45, 46]. Although previous studies generally supported the presence of adverse psychosocial impact and gradual deterioration in mental health during prolonged lockdown [7], the consequences were not necessarily all negative especially during the early phase of the lockdown. For example, Soneson et al. [47] reported that one-third of children and adolescents had improved psychosocial well-being under the first national lockdown in the UK, whereas Branquinho et al. [48] and an earlier report by the UNICEF [49] revealed the potential positive effects on adolescents during initial pandemic lockdown such as temporary respite from schoolwork, increased freedom and autonomy, more time for pleasurable and personal development activities, as well as more parental company. The successful containment of local COVID-19 spread in Mainland China might have also delayed school closure and hence allowed more time for teachers and students to be physically and mentally prepared for class suspensions. Moreover, a high level of trust in government regarding pandemic management, as observed among students in Mainland China, was found associated with better mental health, life satisfaction, and adaptation to containment measures under COVID-19 [50]. Altogether, it seems reasonable to observe a better performance, in particular the overall positive change in psychosocial well-being and the lower level of learning difficulty and loneliness, among students in Mainland China who had not yet been severely affected by the massive local outbreaks and resultant lockdown (that subsequently happened in the later part of 2022) by the time of our data collection.

Apart from the overall performance, the observed significant socioeconomic inequalities in the psychosocial outcomes and factors in all regions echoed the global observations of the more adverse health and social impact of the pandemic on adolescents of poorer socioeconomic background [2–4]. However, there was substantial heterogeneity in terms of the extent of such inequalities across the different regions, with the worst in the Netherlands among the three regions. Such finding could again be attributed in part to the greater inequalities in mental health in countries with more severe COVID-19 outbreaks, as shown in an earlier study by Maffly-Kipp et al. [15]. Relatedly, the high level of unpredictability of daily life under rapidly changing social restrictions on weekly basis, despite being relatively less stringent, to contain COVID-19 spread in the Netherlands could impose an extra psychosocial burden on adolescents, especially for those of lower socioeconomic background living in less economically secured households which are already more vulnerable to job or income loss due to the nature of their employment situations (e.g., low-income jobs, casual work, few opportunity to work from home etc.). Moreover, the greatest extent of socioeconomic inequalities in psychosocial outcomes observed in the Netherlands may be interpreted using some of the hypotheses that Mackenbach formulated for explaining remaining health inequalities in advanced welfare states [19]. First, he hypothesized that inequalities in asset, housing, and other material resources remain despite the income inequalities in these welfare states are relatively low (as in the Netherlands). Second, the shrinking proportion of people of low socioeconomic position is increasingly left behind and therefore relatively more socially disadvantaged as compared to the majority. Third, despite more advanced health improvement in such welfare states, the socioeconomically advantaged have benefited more from these favourable changes due to their better access to resources, information, and services. As a result, the change in social stratification and mobility in the advanced welfare states could have unexpectedly strengthened or created inequalities in health and social conditions. Hence, using Mackenbach’s hypotheses, we speculate that a poor familial socioeconomic background in the Netherlands, as a shrinking minority group, implied extraordinarily high levels of social and environmental stressors and low level of systematic support under the adversities associated with severe local COVID-19 outbreaks. What might have contributed further is the strongly hierarchically ordered high school system in the Netherlands, where the lowest school types are strongly stigmatized and attended mostly by pupils from disadvantaged backgrounds [51].

In contrast to the Netherlands, we observed the lowest extent of socioeconomic inequalities in psychosocial outcomes among students in Mainland China. Given the link between COVID-19 severity and disparities in mental health [15], we initially speculated that the relatively well-controlled pandemic in Mainland China might have alleviated the stressors that otherwise would have disproportionately affected students of poorer socioeconomic background under severe local outbreaks. Nonetheless, the situation appeared to be more complicated than expected as we also found substantial inequalities in psychosocial factors among students in Mainland China despite their better overall outcomes. One of the possible explanations is the unintended consequences of the high overall stringency of COVID-19 containment measures in Mainland China. Previous studies have shown disproportionate negative impact of containment measures (including border control, small-district lockdown, work suspension, and other social distancing measures) on the economic security and daily living routines of both students and their families [52–55]. This may support our findings on the exceptionally high extent of socioeconomic inequalities regarding the impact of family’s financial status, overall worry about COVID-19, and adaptation to social distancing in Mainland China as compared to Hong Kong and the Netherlands. However, the mismatch between the observed inequalities in psychosocial factors and that in psychosocial outcomes in Mainland China was plausibly due to the presence of other protective factors, as suggested by the stress-vulnerability model [56]. Notably, the absolute mean level of resilience and adaptation to social distancing was substantially higher among students in Mainland China without exceptionally high inequality, implying that students of lower socioeconomic position in Mainland China tended to be, on average, more resilient and adaptive than their counterparts in Hong Kong and the Netherlands. As highlighted by Dvorsky et al. [12], the resilience of adolescents plays a crucial role in mitigating or even evading mental health challenges under the pandemic, where a higher level of resilience facilitates successful adaptation, coping, and recovery in the context of the COVID-19-induced psychosocial distress. Hence, the greater overall resilience level and adaptation to social distancing among the socioeconomically disadvantaged students in Mainland China could have enabled a more effective buffering against the impact of COVID-19 stressors on their psychosocial well-being as compared to their counterparts in Hong Kong and the Netherlands. This also resonates with the findings from a recent prospective cohort study in China, which shows that social capital in terms of trust in neighbors, trust in local government officials, and reciprocity was more crucial in longitudinally reducing depression during the pandemic period than before the pandemic [57]. As social capital builds resilience by enabling individuals and communities to support each other during difficult times [58], our findings implied that social capital should also be taken into account for pandemic response and mental health resilience.

In addition to the extent of inequalities, our findings also informed the key intervention entry points for inequality reduction. Specifically, learning difficulty and resilience stood out to explain the observed socioeconomic inequalities in psychosocial well-being among students across the three regions. Therefore, strategies to support remote learning during school closure for the socioeconomically disadvantaged [59, 60], as well as resilience-building programmes for better disaster preparedness [61, 62], should be adopted as a universal approach to improve psychosocial well-being and its socioeconomic inequalities among students. Nonetheless, since the psychosocial factors included in this study could explain only a small proportion of the observed inequalities in the Netherlands, further studies are warranted to explore the context-specific mechanisms behind the disproportionate impact of the pandemic.

The present study has several caveats. First, due to the cross-sectional research design, temporal sequence of the associations could not be established for causal inferences and therefore caution is required when interpreting the mediation pathways. Second, purposive sampling of schools was adopted with unbalanced sample sizes across regions due to the difficulty in random sampling under the pandemic. To this end, we recruited schools of diverse socioeconomic backgrounds to maximize the qualitative generalizability of our sample in each region. Third, the data collection period varied slightly across regions between July 2021 and January 2022; therefore, the study findings should be interpreted with caution as the psychosocial impact of COVID-19 may change over time, and that could have either worsened under prolonged lockdown or waned if students became better adapted to the pandemic-related uncertainty. Fourth, there may be potential clustering effect at regional level as we did not have sufficient number of classes for multi-level analyses within and across regions; instead, we adopted stratified and moderated analyses by regions to explore the potential heterogeneity across regional samples for all analyses. Fifth, the assessment of socioeconomic position, psychosocial outcomes, and associated factors were self-reported, which may be subject to recall bias and social desirability bias. Nonetheless, as our online surveys were anonymous with no personal identifiers being collected, the risks of dishonesty and social desirability are expected to be lower than in other interviewer-assisted surveys [63]. Last, residual effects of socioeconomic position on psychosocial outcomes may exist due to inadequate confounding control and alternative psychosocial factors not being included for analysis in the present study.

To conclude, socioeconomic inequalities in psychosocial well-being were evident among adolescents despite substantial variations across Hong Kong, Mainland China, and the Netherlands under the COVID-19 pandemic. The extent of inequalities in psychosocial outcomes appeared to be greatest in the Netherlands and the lowest in Mainland China, whereas that in psychosocial factors were more apparent in Mainland China than in the other two regions. This cross-regional comparative study allowed us to observe the contextual effects of COVID-19 severity, timeliness, and stringency of containment measures, and the unintended consequences of an advanced welfare system, as well as the differential resilience levels and attitudes towards the government and social distancing measures of students across the three regions that might all have played a role in shaping the impact of socioeconomic disadvantages on psychosocial well-being among adolescents. Further studies are warranted to explore the context-specific mechanisms behind the disproportionate impact of COVID-19 on the psychosocial health inequalities in each region.

## Data Availability

The datasets used and/or analysed during the current study are available from the corresponding author on reasonable request.
